# The Intergenic Type LncRNA (LINC RNA) Faces in Cancer with In Silico Scope and a Directed Lens to LINC00511: A Step toward ncRNA Precision

**DOI:** 10.3390/ncrna9050058

**Published:** 2023-09-25

**Authors:** Shorouk Eldash, Eman F. Sanad, Dina Nada, Nadia M. Hamdy

**Affiliations:** 1 Pharmacology and Biochemistry Department, Faculty of Pharmacy, The British University in Egypt (BUE), El Sherouk, Cairo 11837, Egypt; shorouk.eldash@bue.edu.eg (S.E.);; 2 Biochemistry Department, Faculty of Pharmacy, Ain Shams University, Abassia, Cairo 11566, Egypt

**Keywords:** lncRNA, lincRNA, LINC00511, SNPs, single nucleotide polymorphisms, cancer, prognostic molecular marker, in silico, ncRNA precision

## Abstract

Background: Long intergenic non-coding RNA, is one type of lncRNA, exerting various cellular activities, as does ncRNA, including the regulation of gene expression and chromatin remodeling. The abnormal expression of lincRNAs can induce or suppress carcinogenesis. Main body: LincRNAs can regulate cancer progression through different mechanisms and are considered as potential drug targets. Genetic variations such as single nucleotide polymorphisms (SNPs) in lincRNAs may affect gene expression and messenger ribonucleic acid (mRNA) stability. SNPs in lincRNAs have been found to be associated with different types of cancer, as well. Specifically, LINC00511 has been known to promote the progression of multiple malignancies such as breast cancer, colorectal cancer, lung cancer, hepatocellular carcinoma, and others, making it a promising cancer prognostic molecular marker. Conclusion: LincRNAs have been proved to be associated with different cancer types through various pathways. Herein, we performed a comprehensive literature and in silico databases search listing lncRNAs, lincRNAs including LINC00511, lncRNAs’ SNPs, as well as LINC00511 SNPs in different cancer types, focusing on their role in various cancer types and mechanism(s) of action.

## 1. Introduction

The study of epigenetics, namely, non-protein coding RNAs (ncRNAs), has had significant increased attention recently [1]. ncRNAs are involved in a variety of physiological activities, including the regulation of gene expression and RNA splicing [2]. Long ncRNAs (lncRNA) have a length of more than 200 nucleotides and do not code for proteins [3]; at every stage of gene expression, they act as either an indicator-, decoy-, or scaffold-, and guide-lncRNA [4]. A variety of human disorders have been linked to abnormal lncRNA expression [5]. For example, Nicotinamide nucleotide transhydrogenase-antisense 1 (NNT-AS1) overexpression has been observed in a variety of malignancies, including osteosarcoma, cervical cancer (CC), breast cancer (BC), colorectal cancer (CRC) [6], gastric cancer (GC), Hepatocellular carcinoma (HCC), and non-small cell lung cancer (NSCLC). The biological roles and expression levels of lncRNA transcripts may be affected by variations in lnc gene loci [7].

### 1.1. Long Non-Coding RNAs (LncRNAs)

According to the Genomics Of Long non-coding RNA and Disease Lab GOLD_Lab_ [8] https://www.gold-lab.org/why-lncrnas (accessed on 31 March 2023), the number of protein-coding genes is ~19,000, and until now, <2000 lncRNAs have been investigated; therefore, 98% of lncRNAs are completely uncharacterized, making it worth studying to find new treatment targets, in other words, to find precision prognostic molecular markers. According to their different genetic origins, lncRNAs can be categorized into five groups: sense, antisense, intronic, bidirectional, and intergenic lncRNAs. Sense-lncRNA coincides with the exons of the corresponding protein-coding gene on the sense RNA strand. Antisense lncRNA is derived from the antisense (AS) RNA strand of the protein-coding gene [9]. Intronic lncRNA is the lncRNA that originates from the introns of the protein-coding gene [10]. Moreover, from the promoter of a protein-coding gene, bidirectional lncRNA is transcribed, but in the opposite direction. Finally, long intergenic non-protein coding RNAs (LincRNAs) are the lncRNAs that are situated between two protein-coding genes [11]. RNA polymerase II (Pol II) is primarily responsible for lncRNAs transcription, making them processed in a less efficient manner and more retained in the nucleus rather than the cytoplasm. Nuclear lncRNAs have a role in condensate formation or can be bound to chromatin [12].

### 1.2. LncRNAs and, in Particular, LINC RNA Worth Studying

LincRNAs make more than 50% of lncRNAs [13]. LincRNAs are lncRNAs that are interspersed between coding genes and do not overlap any protein-coding sequences. LincRNAs perform physiological processes like inflammation during infection [14]. LincRNAs exhibit tissue-specific expression [15], being essential for many cellular activities including the control of gene expression [16]. LincRNAs have a pathological role during cancer development [14], when the control of gene expression is perturbed after lincRNA overexpression or mutation.

### 1.3. Review Methodology

A manual online search into two medical e-databases, PUBMED and Google Scholar, for (“lincRNA”) AND (“lincRNA Role in Carcinogenesis”) AND (“lncRNA linc00511”) AND (“lincRNA SNPs”) AND (“future promising biomarkers”) was done in September, 2022. Priority was given to meta-analysis, randomized clinical studies, systematic review, original papers, and narrative reviews, since, but not limited to, 2010.

### 1.4. Review Aim

Introducing lncRNAs and their classification, briefly listing “LincRNAs in different cancer types” after identifying lncRNAs and lincRNAs via in silico databases search, mentioning LincRNAs’ role and mechanism of action in various cancer types, highlighting “LINC00511 in several cancer types”, and finally, listing published documented “Single nucleotide polymorphisms (SNPs) variants of lncRNAs and LINC00511 in different cancer types and SNPs role/mechanism of action influencing cancer risk”.

## 2. Long Intergenic Non-Coding RNAs (LincRNAs) and Their Involvement in Cancer

### 2.1. In Silico Databases Search (accessed on 22 November 2022 and Revised on 31 March 2023)

#### 2.1.1. According to the Database by Ghent University LNCipedia 

Version 5.2 [17] https://lncipedia.org/ in total, there were 127,802 transcripts and 56,946 genes for lncRNA sequence and annotation. The lncRNA class is intergenic and the Sequence Ontology term is lincRNA, where 87 lncRNA transcripts are found (Table 1).

#### 2.1.2. Pseudogenes-Derived LncRNAs (a Hot Area for Research; A Research Gap to Tackle)

Pseudogenes are defective copies of the genes that do not code for proteins [18]. Pseudogenes were considered as junk genes which have no functions; however, recent studies proved that pseudogene-derived lncRNAs have a role in various cancer types through being key regulators at DNA, RNA, or protein levels [19]. LincRNAs that are approved pseudogenes loci according to the National Human Genome Research Institute (NHGRI) grant HUGO gene nomenclature committee (HGNC) https://www.genenames.org/ [20] are four; LINC00265-2P [21], LINC00265-3P [22], LINC00268-2P [23] and LINC00328-2P [24] https://www.genenames.org/tools/search/#!/?query=gene_symbol:linc&rows=20&start=0&fil-ter=document_type:%22gene%22&filter=status:%22Approved%22&filter=locus_group:%22Pseudogene%22 Search (accessed on 22 November 2022 and Revised on 31st March 2023).

### 2.2. What Are LincRNAs?

From the intergenic spaces between two genes, polymerase enzyme II transcribes lincRNAs. The majority of annotated lincRNAs have several exons, cap, and a poly(A) tail that are similar to those of mRNA [25]. LincRNAs have a wide range of functions, including regulating epigenetic changes and regulating gene expression, as well as acting as scaffolds for protein signaling complexes [26]. LincRNA genes differ from mRNA-encoding genes in that they perform crucial roles such as chromatin remodeling and genome architectural remodeling, RNA stabilization or enhancer-associated activity [27], and finally, transcription regulation/control of neighbor genes as well [28].

#### 2.2.1. LincRNA Role in Chromatin Remodeling

**Figure 1:**
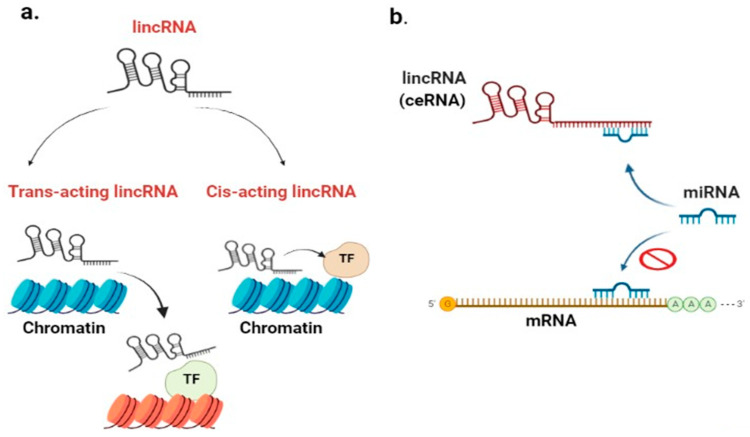
LincRNA role in chromatin remodeling: (**a**) cis-acting lincRNA influences the expression of genes on the same chromosome that are close to its transcriptional location. Contrarily, trans-acting lincRNA can regulate gene expression at distinct loci on a different chromosome, with the involvement of TFs. LincRNA role as competitive endogenous RNA (ceRNA); (**b**) lincRNA competes for miR and acts as micro-RNA sponge; consequently, miR will bind to lincRNA instead of binding to mRNA, leading to an increased mRNA expression after being free. [lincRNA: long intergenic non-coding RNA; ceRNA: competitive endogenous RNA; TF: transcription factor; miR: micro-RNA; mRNA: messenger ribonucleic acid].

LincRNAs control gene expression through interacting with chromatin-modifying complexes to alter the later state [29]. Chromatin-modifying enzymes can be repressive or activating, or these enzymes may occasionally have bivalent domains that perform both activities (repression-activation) [30]. As shown in Figure 1a, lincRNAs can function in a cis-acting manner (cis-acting lincRNA) or trans-acting manner on gene expression. Cis-acting lincRNAs influence the expression of genes on the same chromosome close to their transcriptional location. On the contrary, trans-acting lincRNAs regulate gene expression at distinct, distant loci on a different chromosome [31]. For example, a cis-acting lincRNA HOXA transcript at the distal tip (HOTTIP) induces the expression of the HOXA gene. Trans-acting lincRNA HOX transcript antisense RNA 12 (HOTAIR12) silences HOXD gene as well as genes on other chromosomes [27]. LincRNAs interact with/via transcription factors (TFs) directly or indirectly to drive chromatin-modifying enzymes bound to RNA toward particular genomic locations [32].

#### 2.2.2. LincRNA Role in DNA Damage Repair (DDR)

LincRNAs take part in various stages of the DNA repairing process. LincRNA-p21 regulates apoptosis and cancer cell growth by blocking the translation of the target gene and activating p53 signaling [33] as the lincRNA-p21/dec. downstream target gene/p53 axis. LincRNA-p21 was found to interact with heterogeneous nuclear ribonucleoprotein-K (hnRNP-K), a protein that is directed to the tumor suppressor p53 promoters target genes, leading to transcriptional repression of p53-regulated genes [34]. LINC Regulator Of Reprogramming (Linc-ROR) regulates p53 translation, resulting in driving tumorigenesis in many cancers [35]. As a result of exposure to either exogenous or endogenous environmental stressors, DNA double-strand breaks (DSBs) abrasions occur in both DNA strands, damaging them [36]. LincRNAs have role in DSB repair through two pathways, namely, homologous recombination (HR) and the non-homologous end-joining (NHEJ) pathway. NHEJ involves ligation of the break ends without the need for a homologous template. Whereas in HR, a homologous template sequence is needed [37]. Prostate cancer associated transcript-1 (PCAT-1) is the first lincRNA known to play a role in DSBs repair [38].

#### 2.2.3. LincRNA Role as a Competitive Endogenous RNA (ceRNA)

The interaction between lncRNAs/micro-RNAs/mRNAs makes up a complex regulatory network system, known as the competitive endogenous (ceRNA) network [39]. ceRNAs play a remarkable role in cancer and gene regulation. RNA-induced silencing complex (RISC) maintains this interaction and determines the post transcriptional regulation level of gene expression [40]. As shown in Figure 1b, lincRNAs have the potential to compete for micro-RNA (miR) and behave as a miR sponge. This later process attenuates miR activity and increases the expression of target mRNA genes [39]. LINC00691 is an example of ceRNA that competes for miR-1256 and regulates the expression or suppression of tumorigenicity 5 (ST5), leading to the suppression of sarcoma [41]. LINC00511, overexpressed in BC, has been found to sponge miR-185-3p [42].

#### 2.2.4. LincRNAs Role as Protein Scaffold (PS)

Utilizing scaffolding molecules, which can bring several components together and direct them to enhance their activities, is one method by which the cell can overcome the difficulty of coordinating certain interactions [43]. In the nucleus, scaffold Polycomb repressive complex proteins (PRCP) lincRNA has an impact on the accessibility of chromatin, gene expression, and the structure of the nucleus [44]. Terminal differentiation-induced non-coding RNA (TINCR) lncRNA facilitates the post-transcriptional stability and accumulation of mRNAs that promote epidermal development by scaffolding staufen1, an RNA-binding protein, with the TINCR lincRNA box motif [27].

### 2.3. LincRNAs in Different Types of Cancer

Using RNADisease v4.0; RNA-Disease Repository; RNA-associated diseases, providing RNA-disease analysis, enrichment, and prediction; http://www.rnadisease.org/download for lncRNA-disease information (accessed on 22 November 2022 and revised on 31 March 2023).

#### 2.3.1. LincRNAs List in Different Types of Cancer, Their Role and Mechanism(s) of Action (Table 2)

MALAT1 may represent a potential non-invasive biomarker for HCV-related hepatocellular carcinoma (HCC) prognosis, via sponging miR-204, miR-143, miR-195, miR-490, miR-216b, miR-146-5p [45]. LINC00657 can inhibit glioblastoma (GBM) through sponging miR-190a-3p and the regulation of Phosphatase and tensin homolog (PTEN) expression [46]. Moreover, LINC00707 was found to contribute to glioma cells proliferation, invasion, and migration by sponging miR-613 [47]. Elevated expression of LINC00152 sponges miR-193b-3p to induce phosphorylation and activation of the PI3K signaling pathway and downstream AKT, resulting in tongue squamous cell carcinoma (TSCC) progression [48]. LINC00662 has been discovered to promote prostate cancer tumorigenesis through sponging miR-34a [49]. LINC00657 was found to inhibit CC by sponging miR-20a-5p and the upregulation of RUNX Family Transcription Factor 3 (RUNX3) [50]. LINC01567 can regulate the proliferation of colon cancer stem cells (CSCs) through sponging miR-9, resulting in the modulation of Cyclin D2 (CCND2) and the regulation of aquaporin 3 (AQP3), which can be regulated by the CREB molecule in the cAMP–PKA pathway [51].

Also, LINC00473 contributes to the proliferation and migration of GC by acting as a ceRNA of miR-16-5p [52]. Moreover, LINC00355 can promote the progression of GC by the regulation of the wingless-INT (Wnt)/β-catenin signaling pathway [53]. In addition, Linc01555 contributes to GC cell proliferation through interacting with the Notch signaling pathway [54,55]. HOTAIR is upregulated in Laryngeal squamous cell carcinoma (LSCC), inducing invasiveness, progression, and resistance to apoptosis in LSCC cells through promoting PTEN methylation [56]. Moreover, LINC00673 promotes the progression of lung adenocarcinoma through the activation of the Wnt/B-catenin pathway [57,58]. Again, MALAT-1 contributes to the development of different types of cancer through interaction with Serine/Arginine splicing factors and changing their distribution to nuclear speckle domains [59]. Furthermore, Myocardial Infarction Associated Transcript (MIAT) can promote neuroblastoma by the modulation of MYCN and Paired-like homeobox 2b (PHOX2B) driver genes [60].

**Table 2:** LincRNAs list in different types of cancer, expression level if upregulated or downregulated, sponging-miR, mechanism(s) of action, role in cancer as oncogene or tumor suppressor.

Cancer Type	LincRNA	Expression	Sponging miR-	Mechanism of Action [Ref.]	Role in Cancer	Approach of the Study	Type of Samples Used in the Study
HCC	MALAT-1	Upregulated	204, 143, 195, 490, 216b, 146-5p	Promoting disease progression [45]	Oncogene	Knockdown of MALAT-1	Human blood samples
GBM	LINC00657	Downregulated	190a-3p	Regulation of PTEN expression [46]	Tumor suppressor	Overexpression of LINC00657	Human GBM tissues vs. adjacent normal tissues and GBM cell lines U-87 MG, LN-18, U-118 MG vs. astrocyte HA1800
	LINC00707	Upregulated	613	Promotes progression, migration and invasion of glioma cells [47]	Oncogene	Knockdown of LINC00707	Human Glioma tissues vs. adjacent normal tissues and glioma cell lines U87, U251, SHG-44, A172, T98G vs. normal astrocyte cell lines NHA, human embryonic kidney cell line HEK-293
TSCC	LINC00152	Upregulated	193b-3p	PI3K signaling pathway activation and downstream AKT enhancing cell cycle progression, tumor migration, invasion [48]	Oncogene	Knockdown of LINC00152	Human TSCC tissues vs. adjacent normal tissues and cell lines SCC-9, CAL-27
Prostate	LINC00662	Upregulated	34a	Promotes cancer progression [49]	Oncogene	Knockdown of LINC00662	Human Prostate cancer tissues vs normal tissues and prostate cancer cells DU145, 22RV1, PC-3, and LNCaP vs. normal prostate epithelial cells WPMY-1
CC	LINC00657	Downregulated	20a-5p	Upregulation of RUNX3 that targets DR5 leading to activation of NK cells [50]	Tumor suppressor	Overexpression of LINC00657	Human CC tissues vs normal tissues and CC cell lines SiHa, HeLa, C33A, Caski vs. normal cervical squamous cell line Ect1/E6E7
Colon CSCs	LINC01567	Upregulated	9	CCND2 modulation and AQP3 regulation CREB/cAMP–PKA and proliferation and tumorigenesis regulation of colon CSCs [51]	Oncogene	Knockdown of LINC01567	Human Colon cancer tissues vs. normal tissues
GC	LINC00473	Upregulated	16-5p	Modulating CCND2 expression, Promoting progression of GC and migration [52]	Oncogene	Knockdown of LINC00473	Human GC tissues vs. normal tissues and GC cell lines BGC823, AGS, MKN-45, NCI-N87, SGC7901 vs GES-1 and female BALB/c-nude mice for implantation
	LINC00355	Upregulated	-	Regulating Wnt/β-catenin, promoting progression and inhibition of apoptosis [53]	Oncogene	Knockdown of LINC00355	Human GC tissues vs normal tissues and GC cell lines BGC-823, MGC-803, AGS, SGC-7901 vs normal gastric epithelial cells GES-1
	LINC01555	Upregulated	-	Interacting with Notch signalling pathway for progression of GC [54,55]	Oncogene	Knockdown of LINC01555	Human GC tissues vs para-carcinoma tissues and GC cell lines MGC803, MKN45, BSG823, SGC7901, vs normal human gastric mucosal epithelial cell GES-l
LSCC	HOTAIR	Upregulated	-	Promoting PTEN methylation, progression, invasiveness, resistance to apoptosis [56]	Oncogene	Knockdown of HOTAIR	Human LSCC tissues vs adjacent normal tissues and and mice BALB/c for implantation
Lung adenocarcinoma	LINC00673	Upregulated	-	Activation of Wnt/B-catenin for progression of the disease [57,58]	Oncogene	Knockdown of LINC00673	Human lung adenocarcinoma cell lines HCC827, NCI-H1650, A549, NCI-H596, NCI-H1975, NCI-H1299, SK-LU-1, NCI-H358, NCI-H2009, HCC4006, NCI-H2030, PC9, and nude mice for implantation
Various cancer types	MALAT-1	Upregulated	-	Interaction with Serine/Arginine splicing factors, changing distribution to nuclear speckle domains, promoting progression of the disease [59]	Oncogene	Knockdown of MALAT-1	Human HeLa cells
Neuro-blastoma	MIAT	Upregulated	-	Modulation of MYCN and PHOX2B driver genes leading to progression of the disease [60]	Oncogene	Knockdown of MIAT	RNA sequencing data analysis
BC	Linc-ROR	Upregulated	205, 145	EMT induction and promoting metastasis [61]	Oncogene	Knockdown of Linc-ROR	Human BC tissues vs adjacent normal tissues and MCF10A, MDA-MB-231, BT549, BT474, MDA-MB-436, MDA-MB-435, HEK 293 and immunodeficient nude mice for implantation
	LincRNA-BC2	Upregulated	-	Interacting with BRCA1 and BRCA2 [62]	Oncogene	Knockdown of LincRNA-BC2	Human BC tissues vs adjacent normal tissues
TNBC	LINC00299	Hyper-methylated	-	Hypermethylation [63]	Oncogene	Knockdown of LINC00299	Human blood samples
BC	LINC00641	Downregulated	194-5p	Inhibition of cell growth, invasion, migration [64]	Tumor suppressor	Overexpression of LINC00641	Human BC tissues vs adjacent normal tissues and BCAP-37, MDA-MB-453, UACC-812, MCF-7, MDA-MB-231 vs normal breast epithelial cell line MCF-10A
TNBC	LINC00993	Downregulated	-	Generating G0/G1 arrest and regulation of p21 and p53 genes [65]	Tumor suppressor	Overexpression of LINC00993	Human BC cell lines MDA-MB-231, BT-549 and female BALB/c nude mice for implantation
BC	LINC00885	Upregulated	-	EGFR, EREG, FOXM1 and TP53 activation, progression of early stage BC [66]	Oncogene	Knockdown of LINC00885	Human BC cell lines MCF10 DCIS.COM, MDA-MB-231, MCF7, T47D vs normal breast epithelial cell lines MCF10A, 184A1
	Linc-APOC1P1-3	Upregulated	-	Binding tubulin to decrease α-tubulin acetylation and inactivate caspase-3, BC progression, prevention of BC cells apoptosis [67]	Oncogene	Knockdown of Linc-APOC1P1-3	Human BC tissues vs normal tissues
	Linc-HOTAIR	Upregulated	-	Interaction with PRC2 and Promoting BC metastasis [68]	Oncogene	Knockdown of Linc-HOTAIR	Human BC tissues vs normal tissues, human cell lines MDA-MB-231, SK-BR-3, MCF-10A, MCF-7, HCC1954, T47D, MDA-MB-453, H16N2 and nude mice for implantation
	LINC00657	Upregulated	590-3p	GOLPH3 upregulation leading to invasion, migration, proliferation and inhibition of apoptosis of BC cells [69]	Oncogene	Knockdown of LINC00657	Human BC tissues vs adjacent normal tissues and human BC cell lines MCF-7, MDA-MB-231, T47D, BT-549 vs normal breast epithelial cell line MCF-10A
	LINC00511	Upregulated	185-3p	- [42]	Oncogenic	Knockdown of LINC00511	Human blood samples
	LINC00460	Upregulated	320a	MAL2 upregulation and promoting cancer cell proliferation and migration [70]	Oncogene	Knockdown of LINC00460	MDA-MB-231, BT-549 cells
	LINC00922	Upregulated	-	Promoting NKD2 methylation, promoting tumorigenesis, invasion, metastasis and regulation EMT [71]	Oncogene	Knockdown of LINC00922	Human BC tissues vs adjacent normal tissues and human MCF-7, MDA-MB-231, SK-BR3, MCF10A
CRC	LINC01088	Upregulated	548b-5p and 548c-5p	G3BP1 expression upregulation, enhancing CRC progression [72]	Oncogene	Knockdown of LINC01088	Human CRC tissues vs para-cancerous tissues, human CRC cell lines, colonic epithelial cells, and mice for implantation

[MALAT1: Metastasis Associated Lung Adenocarcinoma Transcript 1; PTEN: Phosphatase and tensin homolog; TSCC: Tongue Squamous Cell Carcinoma; SCC: Squamous Cell Carcinoma; CC: Cervical Cancer; RUNX3: RUNX Family Transcription Factor 3;CSCs: Cancer Stem Cells; CCND2: Cyclin D2; AQP3: aquaporin; 3GC: Gastric Cancer; ceRNA: competing endogenous RNA; Wnt: Wingless/Integrated; HOTAIR:HOX transcript antisense RNA; LSCC: laryngeal squamous cell carcinoma; MIAT: Myocardial Infarction Associated Transcript; PHOX2B: Paired-like homeobox 2b; Linc-ROR: Long Intergenic Non-Protein Coding RNA, Regulator Of Reprogramming; BC: Breast Cancer; EMT: Epithelial–Mesenchymal Transition; LincRNA-BC2: long intergenic non-coding RNA-Breast Cancer 2; BRCA1: Breast Cancer 1; BRCA2: Breast Cancer 2; RPISeq: RNA-Protein Interaction; TNBC: Triple Negative Breast Cancer; EGFR: epidermal growth factor receptor, EREG: epiregulin; FOXM1: forkhead box M1; Linc-APOC1P1-3: long intergenic non-coding RNA APOC1P1-3; PRC2: Polycomb Repressive Complex 2; GOLPH3: Golgi phosphoprotein 3; MAL2: Myelin And Lymphocyte protein 2; NKD2: Naked cuticle homolog 2; G3BP1: G3BP Stress Granule Assembly Factor 1].

#### 2.3.2. LincRNAs List in Breast Cancer (BC)

Linc-ROR could promote BC metastasis through sponging miR-205, miR-145, resulting in epithelial–mesenchymal transition (EMT) induction [61]. LincRNA-BC2 is upregulated in BC, with interaction with BC associated protein antigens 1 and 2 (BRCA1 and BRCA2), which are predicted by RNA-Protein Interaction Prediction (RPISeq) [62]. Moreover, LINC00299 was found to be a promising biomarker for triple negative BC (TNBC) by its hypermethylation [63]. It has been reported that LINC00641 can be a target for BC treatment, since it inhibits proliferation, invasion, and migration of BC cells via sponging miR-194-5p [64]. LINC00993 suppresses the TNBC growth *in vitro* and *in vivo* by generating G0/G1 arrest and regulation of the genes related to cell cycle such as p21 and p53 [65]. LINC00885 was reported to act as ceRNA and regulate BC cells growth. In addition, LINC00885 is associated with early stage BC through the activation of epidermal growth factor receptor (EGFR), epiregulin (EREG), and forkhead box M1 (FOXM1) pathways and signaling pathways linked to TP53 signaling [66]. Liao et al. have found that Linc-APOC1P1-3 expression induces proliferation and inhibits apoptosis of BC cells via binding tubulin to decrease α-tubulin acetylation and, therefore, inactivates caspase-3 [67]. Researchers pointed to Linc-HOTAIR interaction with Polycomb Repressive Complex 2 (PRC2) promoting BC metastasis [68]. LINC00657 was found to play a role in the biological behavior of BC such as invasion, migration, proliferation, and apoptosis. It can sponge miR-590-3p and upregulates Golgi Phosphoprotein 3 (GOLPH3) [69]. LINC00511 was found by our group to be overexpressed in BC patient’s blood, with sponging miR-185-3p, and was considered as an early diagnostic biomarker for BC [42]. BC cell migration and proliferation was promoted by LINC00460 via sponging miR-320a and upregulation of MAL2 [70]. In addition, LINC00922 could induce BC invasion, metastasis, progression, and regulation of EMT, by NKD2 methylation [71]. All lincRNAs roles and mechanisms of action in BC are mentioned in Table 2. LINC01088 was found to bind to miR-548b-5p and miR-548c-5p to upregulate G3BP1 expression, resulting in the enhancement of CRC progression with immune scape and finally, changing cancer cell phenotypes [72].

As summarized in Table 2 “Long intergenic non-coding RNAs (lincRNAs) and their involvement in cancer”, LincRNAs have several important functions, such as chromatin remodeling, DNA damage/repair, and acting as ceRNA or protein scaffold. There are various lincRNAs that have a role in different types of cancer, leading to an increase or decrease in its risk with different mechanisms.

## 3. LINC00511 and Its Contribution in Different Cancer Types

### 3.1. LINC00511 In Silico Info (Accessed on 25 November 2022 and Revised on 31 March 2023)

**Figure 2:**
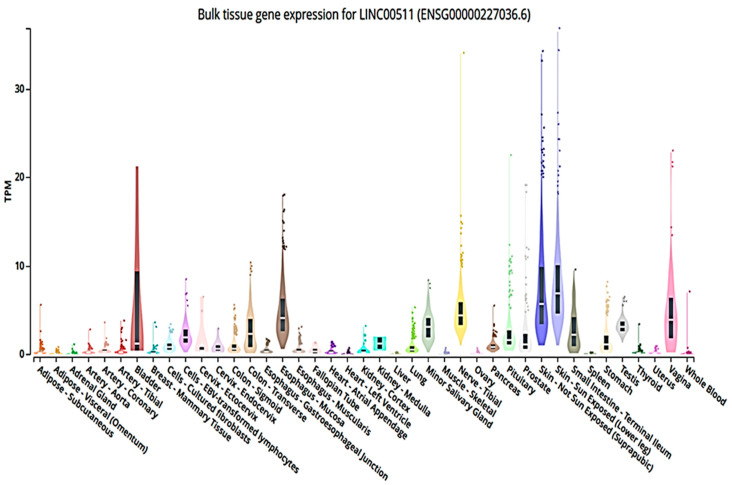
Tissue gene expression graph for LINC00511 in some tissue sites. The expression graph of LINC00511 is represented in transcripts per million (TPM), as TPM is an accurate statistic used when calculating gene expression comparisons across samples. Here, Figure 2 represents the expression values of LINC00511 in non-diseased tissue sites samples collected from 1000 individuals for molecular assays. As shown, the highest expression of LINC00511 was found in the sun-exposed skin tissues from GTEx Analysis https://www.gtexportal.org/home/gene/LINC00511 (accessed on 25 November 2022 and revised on 31 March 2023).

The International Cancer Genome Consortium (ICGC) data portal [73,74] identified LINC00511 gene as 2265-bp, which is localized on chromosome 17q24.3 with five exons https://dcc.icgc.org/genes/ENSG00000227036 [75]. Bulk tissue gene expression for LINC00511 (ENSG00000227036.6) from the Genotype-Tissue Expression (GTEx) project helped to study tissue-specific gene expression and regulation, where 54 non-diseased tissue sites samples were collected from 1000 individuals for molecular assays. GTEx Analysis Release V8 (dbGaP Accession phs000424.v8.p2) https://www.gtexportal.org/home/gene/LINC00511. Figure 2 shows the LINC00511 expression in different tissues. The highest expression of LINC00511 was found in the sun-exposed skin tissues.

### 3.2. LINC00511 in Cancer

LINC00511 was first discovered by Cabanski et al. in 2015 [76]. LINC00511 is dysregulated in multiple malignancies including glioma, BC, ovarian cancer, CC, osteosarcoma, HCC, lung cancer, TSCC, renal cell cancer, papillary thyroid carcinoma, pancreatic cancer, GC [77], and CRC [78]. This dysregulation has a role in facilitating the prognosis of cancer [79]. First, LINC00511 sponge miRNAs and alter the expression of their targets. Second, through interacting with enzymes and TFs associated to DNA methylation, LINC00511 can control tumor suppressors or oncogenes to promote tumorigenesis [77]. Additionally, LINC00511 promotes cell proliferation, cell cycle progression, tumorigenesis, invasion, and metastasis [75]. According to Lu et al., LINC00511 can promote tumor growth and metastasis and induce stemness in malignancies. In order to prevent cancer metastasis, it may be possible to employ LINC00511-modifying modalities [80]. Role of LINC00511 Expression in Different Types of Cancer (Table 3).

#### 3.2.1. LINC00511 Role in Colorectal Cancer (CRC)

Hypoxia Inducible Factor 1 (HIF-1) could activate LINC00511, to sponge miR-153-5p in CRC cells. MiR-153-5p targets HIF-1’s 3-UTR, forming a positive feedback loop of HIF-1/LINC00511/miR-153-5p in CRC cells [78]. LINC00511 interacts with enhancer of zeste homolog 2 (EZH2), leading to downregulation of interleukin-24 (IL-24) expression. Lu et al. found Hepatocyte Nuclear Factor4 (HNF4) could promote LINC00511 transcription to accelerate cancer progression; therefore, the LINC00511/EZH2/IL-24 axis is a potential therapeutic target [81]. Hu et al. found LINC00511 to sponge miR-29c-3p, leading to upregulation of Nuclear Factor I A (NFIA) with subsequent progression of CRC [82]. Moreover, LINC00511 promotes CRC progression by suppressing miRNA-625-5p to enhance WEE1 protein [83].

#### 3.2.2. LINC00511 Role in Lung Cancer

Zhu et al. have found that LINC00511 promotes NSCLC through binding to lysine-specific demethylase 1 (LSD1) and EZH2, resulting in Large Tumor Suppressor Kinase 2 (LATS2) and KLF Transcription Factor 2 (KLF2) genes inhibition [84]. LINC00511 mediates oncogenesis of NSCLC via binding EZH2, silencing p57 expression. LINC00511 knockdown inhibited carcinogenesis in vivo and slowed cell proliferation via promoted apoptosis in vitro [85]. In addition, LINC00511 enhances the progression of lung adenocarcinoma by sponging miR-625-5p and regulating Pyruvate kinase M2 (PKM2) expression [86]. Zhang et al. have reported that LINC00511 promotes lung cancer progression by binding to miR-195-5p and upregulating glucosaminyl (N-acetyl) transferase 3 (GCNT3) [87]. LINC00511 can target miR-625-5p/GSPT, contributing to NSCLC proliferation and invasion [88]. However, inhibiting miR-150-5p and activating Transcriptional Adaptor 1 (TADA1), now LINC00511 can enhance the proliferation and migration of lung squamous cell carcinoma [89].

#### 3.2.3. LINC00511 Role in Cervical Cancer (CC)

LINC00511 promotes CC by upregulating phospholipase D1 (PLD1) expression through transcription factor retinoic X receptor alpha (RXRA). Inhibition of LINC00511 induces apoptosis and decreases the progression of CC [90]. Mao et al. have found that downregulation of LINC00511 in CC cells increased sensitivity to paclitaxel, lowered cancer cell viability, proliferation, and induced apoptosis, resulting in CC recurrence prevention through regulating B-cell lymphoma 2 (Bcl-2), Bcl-2 Associated X-protein (Bax), metalloproteinases 2 and 9, multidrug resistance protein 1 (MRP1), P-glycoprotein, and cleaved caspase-3 [91]. LINC00511 can sponge miR-324-5p with regulation of the DRAM1 axis [92]. Moreover, LINC00511 enhances the proliferation and progression of CC through regulation of the miR-497-5p/MAPK1 axis [93].

#### 3.2.4. LINC00511 Role in Gastric Cancer (GC)

LINC00511 promotes GC cells tumorigenesis and stemness by sponging miR-195-5p, then elevating SRY-box transcription factor 4 (SOX4), followed by repressing PTEN, which will activate the PI3K/AKT pathway via recruited EZH2 [94]. Moreover, LINC00511 increases GC cells proliferation by sponging miR-515-5p [95]. LINC00511 enhances GC cells growth by sponging miR-124-3p and regulating the miR-124-3p/PDK4 axis [96]. LINC00511 promotes gastric tumorigenesis by sponging miR-625-5p and targeting the nuclear factor 1/X gene (NFIX) [97].

#### 3.2.5. LINC00511 Role in Pancreatic Cancer (PC)

LINC00511 competitively endogenously inhibits hsa-miR-29b-3p activity to upregulate vascular endothelial growth factor A (VEGFA), promoting pancreatic ductal adenocarcinoma (PDAC) stemness. Therefore, LINC00511 is considered as a promising biomarker that can be used to predict PDAC patients prognosis following surgery, and could be a therapeutic target [98].

#### 3.2.6. LINC00511 Role in Hepatocellular Cancer (HCC)

LINC00511 could competitively interact with miR-424 to promote HCC proliferation and metastasis [99]. LINC00511 promotes HCC development by competing with miR-195 and positively correlating with Eyes absent homolog 1 (EYA1) [100]. Moreover, LINC00511 accelerate HCC progression, acting as a prognostic biomarker for the disease. However, it has an adverse interaction with miRNA-29c in HCC [101]. It is noteworthy to mention that LINC00511 promotes HCC invasion via affecting exosome secretion and invadopodia formation [102].

#### 3.2.7. LINC00511 Role in Glioblastoma (GBM)

Du et al. discovered LINC00511 to act as a ceRNA sponging miR-524-5p, indirectly controlling the Y box binding protein 1 (YB1), boosting Zinc finger E-box-binding homeobox 1 (ZEB1) expression. This enhanced LINC00511 expression in a reverse way constructing LINC00511/miR-524-5p/YB1/ZEB1 positive feedback loop that encourage GBM cell migration and invasion [103]. Via targeting miR-15a-5p/AE Binding Protein 1 (AEBP1) axis, LINC00511 knockdown can prevent glioma cell carcinoma development [104]. LINC00511 sponges miR-126-5p and activate Wnt/β-catenin signaling, facilitating Temozolomide resistance of GBM [105].

#### 3.2.8. LINC00511 Role in Osteosarcoma (OS)

LINC00511 induces OS via sponging miR-618 and boosting Maelstrom Spermatogenic Transposon Silencer (MAEL) expression [106]. LINC00511 sponges miR-185-3p leading to E2F transcription factor 1 (E2F1) expression regulation and promoting OS [107], or sponging miR-765, which promotes apurinic/apyrimidinic endonuclease 1 (APE1) in OS cells [108]. In contrast, another study demonstrated that LINC00511 is downregulated in OS, inhibits cell proliferation and increases apoptosis of OS cells [109].

#### 3.2.9. LINC00511 Role in Different Types of Breast Cancer (BC)

It has been reported that the LINC00511/miR-150/MMP13 axis contributes to BC proliferation and migration. LINC00511 has the ability to sponge miR-150, leading to regulation of expression of Matrix Metallopeptidase 13 (MMP13) and promoting cell migration [110]. DNA hypomethylation induces LINC00511 expression and LINC00511 promotes BC growth by upregulating Wnt family member 10A (Wnt10A), E2F transcription factor 2 (E2F2), Transforming growth factor-alpha (TGF-A), and MET [111]. The LINC00511/miR-185-3p/E2F1/Nanog axis promotes the BC cells growth, via sponging miR-185-3p and target E2F1 protein that binds with the Nanog promoter region to activate its transcription [80]. LINC00511 has been proved to increase the expression of BC cells as well as the transcriptional control of downstream genes through an elevated LINC00511/miR-185-3p axis. Therefore, LINC00511 can be considered a marker for BC early diagnosis [42]. LINC00511 is a TNBC-specific lncRNA that functions as an oncogene to control tumor metastasis and prognosis [112]. LINC00511 enhances ER-negative BC cell growth by altering cell proliferation and apoptosis by accelerating the G1/S transition and suppressing apoptosis [113]. Zhang et al. has proved that LINC00511 downregulation enhances paclitaxel cytotoxicity in BC cells by acting as a miR-29c molecular sponge [114]. Moreover, inhibition of LINC00511 reduces its competitive binding to miR185, leading to higher STXBP4 expression and better radiation response in BC [115].

As well summarized in Table 3 “LINC00511 and its contribution in different cancer types”, different studies have proved that LINC00511 is upregulated in various types of cancer. For example, in CRC, LINC00511 promotes the progression of the disease through sponging miR-153-5p, miR-29-3p, and miR-625-5p. In addition, LINC00511 induces the proliferation of lung cancer by sponging miR-625-5p, miR-195-5p, and miR-150-5p. The progression of CC can be promoted by LINC00511 through sponging miR-324-5p and miR-497-5p. LINC00511 can stimulate GC tumorigenesis by sponging miR-195-5p, miR-515-p, miR-124-3p, and miR-625-5p. Moreover, LINC00511 can induce BC oncogenesis through sponging miR-150, miR-185, miR-185-3p, and miR-29, in addition to other discussed types of cancer that can be induced by LINC00511. Although different studies have reported that LINC00511 is upregulated in OS, one study has proved that it is downregulated in OS and inhibits cell proliferation.

## 4. LncRNAs SNPs in Different Cancer Types and Their Mechanism of Action

Unveiling the relationship between lncRNAs SNPs or specifically, LINC RNA SNPs and disease mechanism(s) is an important research gap to investigate, as future prospective, to relate cancer incidence or progression/remission to specific variants. This will be a step toward ncRNA precision, fulfilling big pharma’s shift toward targeting RNA for treatment instead of DNA or classical cancer-hallmarks. In the human genome, Over 10 million SNPs have been identified resulting in gene variants, which change the cell’s protein production machinery [116]. SNPs can affect RNA-RNA interaction (lncRNA-miRNA interactions) through a ceRNA mechanism, where lncRNA could competitively bind miRNAs [117]. It has been reported that SNPs within lncRNA transcripts can impact the structure and function of lncRNA, whereas SNPs in an lncRNA’s promoter region might affect its expression level [116]. Furthermore, lncRNAs SNPs that change the structure of the lncRNA influence the interaction between the RNA Binding Proteins (RBPs) and lncRNA, resulting in the regulation of several biological pathways [118]. It has been found that lincRNA SNPs in exon loci may alter the secondary structure of the lincRNA. For example, SNP rs1456315 G/A in lincRNA PRNCR1alters its secondary structure and hence, the conformation and stability of lincRNA, even causing changes in its interacting partners [117].

### 4.1. LncRNAs SNPs List in Breast Cancer, Their Role and Mechanism of Action (Table 4)

CDKN2B-AS1, also named ANRIL, is an lncRNA that can interfere with the expression of neighboring genes, control cell proliferation and apoptosis, and is upregulated in BC. Researchers proved that SNP rs310965215 in ANRIL altered cells’ ability to proliferate, invade, and migrate by sponging miR-4440 [119]. Another study demonstrated that SNPs rs1333045, rs1333048, rs4977574, and rs10757278 in ANRIL increase BC risk [120]. However, MALAT1 SNPs (rs3200401, rs619586, and rs7927113) have an association with BC susceptibility. Fortunately, genotypes AG and AG + GG of MALAT1 SNP rs619586 protect against BC, and CT of rs3200401 reduces BC risk [121]. Growth Arrest Specific 5 (GAS5) is a tumor suppressor and is downregulated in many cancer types including BC [122]. The GAS5 SNP rs145204276 del allele may inhibit BC development by increasing the promoter activity via binding to the TF specificity protein 1 [123]. Cancer susceptibility candidate 15 (CASC15) is a very active lncRNA in silico and is found on chromosome 6p22.3. An interaction between lncRNA CASC15 polymorphisms and susceptibility of BC has been found; rs7740084 and rs1928168 reduced BC risk, whereas, there is a correlation between rs9393266 and BC risk [124]. Similarly, HOTAIR SNP rs920778 elevates BC risk and might interact with the clinical reproductive factors [125]. MiR2052HG rs34841297 regulates miR-4456 expression, which alters BC cells proliferation and invasion, increasing BC susceptibility [126]. LINC00520 is found on human chromosome 14q22.3 [127] and is upregulated in various tumors including LSCC, nasopharyngeal carcinoma, and renal cell carcinoma. Increased TNBC susceptibility may be exploited by LINC00520 SNP rs8012083 [128]. Also, SNP rs527616 in lncRNA AQP4-AS1 increases BC susceptibility [129]. SOX2OT is an lncRNA located in the SOX2 gene in the intronic region. Via affecting the expression of SOX2OT, lncRNA SOX2OT SNP rs9839776 increases BC risk [130]. Again, lncRNA H19 SNPs (rs3741219, rs217727, and rs2839698) increase BC risk, whereas rs3741216 decreased it [131]. SRA is an lncRNA upregulated in BC and its expression correlates with levels of ER and PR. SNP rs10463297 in lncRNA SRA increased BC risk through affecting SRA mRNA expression [132]. SNPs rs11657109, rs17780195, and rs9906859 in LINC00511 may protect against BC, being related to LINC00511 secondary structure and expression [133]. Our group is currently investigating LINC00511 SNPs in BC as well as CRC patients’ blood samples (publication in progress). LincRNA-ROR SNP rs4801078 was correlated to BC risk, being affected by the interplay between linc-ROR SNPs and reproductive factors [134].

**Table 4:** LncRNAs SNPs lists in Breast Cancer (BC), their mechanism of action and role in BC.

LncRNA List	SNPs List	Mechanism of Action [Ref.]	Role in BC	Type of Samples Used in the Study
CDKN2B-AS1; ANRIL	rs310965215	Sponging miR-4440 [117]	Cells’ altered ability to proliferate, invade, migrate	Extracted DNA from human blood samples
	rs1333045, rs1333048, rs4977574, and rs10757278	- [120]	Increased risk	
MALAT1	rs7927113	- [121]	Association with BC susceptibility; AG, AG + GG	
	rs619586	- [121]	Protects against	
	rs3200401	- [121]	Reduces risk	
GAS5	rs145204276	Increasing promoter activity, binding TF specificity protein 1, raise GAS5 [123]	Inhibition of BC development	
CASC15	rs7740084, rs1928168	- [122]	Reduce risk	
	rs9393266	- [124]	Correlated to risk	
HOTAIR	rs920778	Interaction with reproductive factors [125]	Elevation of risk	
MIR2052HG	rs34841297	Regulation of miR-4456 expression [126]	Increased susceptibility	
LINC00520	rs8012083	- [128]	Increased TNBC susceptibility	
AQP4-AS1	rs527616	- [129]	Increased susceptibility	DNA extracted from human blood samples and BC tissues vs. normal tissues
SOX2OT	rs9839776	Influencing SOX2OT expression [130]	Increases risk and related to onset	Extracted DNA from human blood samples
H19	rs3741219, rs217727, rs2839698	- [128]	Increased risk	
	rs3741216	- [131]	Decreased risk	
SRA	rs10463297	Affecting SRA mRNA expression [132]	Increased risk	
LINC00511	rs11657109, rs17780195, rs9906859	- [133]	Protection	
Linc-ROR	rs4801078	Interplay with reproductive factors [134]	Increased risk	

[CDKN2B-AS1: cyclin-dependent kinase inhibitor 2B antisense RNA 1, ANRIL: antisense non-coding RNA in the INK4 locus; BC: Breast Cancer; ER: Estrogen Receptor; PR: Progesterone Receptor; MALAT1: Metastasis Associated Lung Adenocarcinoma Transcript 1; GAS5: Growth Arrest Specific 5; CASC15: Cancer Susceptibility 15; HOTAIR: HOX transcript antisense RNA; PCAT1: Prostate Cancer Associated Transcript 1; MIR2052HG: MIR2052 Host Gene; LINC00520: Long Intergenic Non-Protein Coding RNA 520; TNBC: Triple Negative Breast Cancer; AQP4-AS1: AQP4 Antisense RNA 1; SOX2OT: SOX2 overlapping transcript; SRA: steroid receptor RNA activator; mRNA: messenger RNA; Linc-ROR: Long Intergenic Non-Protein Coding RNA, Regulator Of Reprogramming].

### 4.2. LncRNAs SNPs List in Lung Cancer, Their Role and Mechanism of Action (Table 5)

NEAT1 is a lncRNA located on chromosome 11q13.1, and is a component of paraspeckles. As seen in Table 5, SNP rs2239895 in lncRNA NEAT1 increases the risk of lung squamous cell carcinoma [135]. CCAT1 is located on chromosome 8q24.21 and is overexpressed in several tumors such as GC, CRC, and HCC. SNP rs1948915 in lncRNA CCAT1 is associated with decreased lung cancer susceptibility, in the study’s female population cohort [136]. Researchers have found that SNP rs219741 in lncRNA LOC105369301 elevates the risk of NSCLC, while SNP rs498238 in lncRNA LINC01833 and SNP rs16901995 in lnc-NDUFS6-5:5 all reduce the NSCLC risk [137]. Compared to people with the homozygous wild AA genotype/heterozygote GA genotype, those with the homozygous GG genotype SNP rs7248320 in lncRNA AC008392.1 had a lower chance of developing NSCLC [138]. Researchers have reached a point that SNP rs4759314 (AG genotype) in lncRNA HOTAIR can increase the risk of lung cancer development, while SNP rs12826786 (“CT” and “CT + TT” genotypes) decreased this risk [139].

Moreover, SNPs rs920778 and rs1899663 in lncRNA HOTAIR have been found to increase lung cancer susceptibility [140]. Epidermal growth factor receptor (EGFR) wild type lung adenocarcinoma, the GAS5 SNP rs145204276, may aid in tumor stage, distal metastases, and lymph node metastasis prediction [141]. PRNCR1 is an lncRNA located on chromosome 8q24.21 and is a popular oncogene in prostate cancer [142]. A study has found that the PRNCR1 SNP rs1456315 T allele compared with the C allele and the lncRNA CCAT2 SNP rs6983267 G allele, compared with the T allele, increased lung cancer risk. These SNPs affect the lncRNA secondary structure as well as the miRNAs target [143]. There has been an association between SNP rs3200401 in lncRNA MALAT1 and susceptibility of lung squamous cell carcinoma and NSCLC, via altered MALAT1’s structural properties and downstream genes contributing to the formation and progression of cancer [144]. HOXA11-AS is a lncRNA whose ectopic expression plays important roles in different cancer types. SNP rs17427875 (T allele) in HOXA11-AS increases the risk of lung adenocarcinoma, whereas SNP rs11564004 (G allele) plays a protective role, with TFs as mediators [145]. SNP rs217727 (A/A homozygous genotype) in lncRNA H19 is associated with elevated lung cancer risk, particularly adenocarcinoma and squamous cell carcinoma [146]. LncRNA LOC146880 is upregulated in NSCLC and is associated with poor prognosis of the disease. The A allele in rs140618127 SNP in lncRNA LOC146880 decreases NSCLC risk. LOC146880 offers microRNA miR-539-5p an alternative binding site, altering ENO1 phosphorylation with PI3K and Akt pathway’s activation [147].

**Table 5:** LncRNAs SNPs list in lung cancer, their mechanism of action and role in lung cancer.

LncRNA List	SNPs List	Mechanism of Action [Ref.]	Role in Lung Cancer	Type of Samples Used in the Study
NEAT1	rs2239895	- [135]	Increased carcinoma risk	Extracted DNA from human blood samples
CCAT1	rs1948915	- [136]	Decreased cancer in females’	
LOC105369301	rs219741	- [137]	Elevated risk	
LINC01833	rs498238	- [137]	Elevated risk	
lnc-NDUFS6-5:5	rs16901995	- [137]	Reduced risk	
AC008392.1	rs7248320	- [138]	Reduced risk in GG genotype	
HOTAIR	rs4759314	- [139]	Increases cancer risk	
	rs12826786	- [137]	“CT” and “CT + TT” decreases risk	
HOTAIR	rs920778 and rs1899663	- [140]	Increased susceptibility	
GAS5	rs145204276	- [141]	Aiding in tumor stage, distal metastases, LN metastasis prediction, in EGFR wild type patients	
PRNCR1	rs1456315	Affecting lncRNA secondary structure and target miRNAs [143]	Increased risk in patients with T allele	
CCAT2	rs6983267	Affecting the secondary structure of lncRNA and target of miRNAs [143]	Increased risk of lung cancer in patients with G allele	
MALAT1	rs3200401	MALAT1’s structural properties alterationand cancer genes expression [144]	Increased susceptibility	
HOXA11-AS	rs17427875	Associating with TFs [145]	(T allele) increases risk	
	rs11564004	- [143]	(G allele) play a protective role	
H19	rs217727	- [146]	Elevated risk in A/A homozygous	
LOC146880	rs140618127	miR-539-5p alternative binding site, ENO1 phosphorylation, PI3K/Akt activation [147]	Decreased risk	Human NSCLC tissues vs. adjacent normal tissues and human NSCLC cell lines A549, PC9 vs. human lung epithelial BEAS-2B cells

[NEAT1: Nuclear Enriched Abundant Transcript 1; CCAT1: Colon Cancer Associated Transcript 1; NSCLC: Non-Small Cell Lung Cancer; HOTAIR: HOX transcript antisense RNA; GAS5: Growth Arrest Specific 5; EGFR: Epidermal Growth Factor Receptor; PRNCR1: Prostate Cancer Associated Non-Coding RNA 1; lncRNA: long non coding RNA; CCAT2: Colon Cancer Associated Transcript 2; MALAT1: Metastasis Associated Lung Adenocarcinoma Transcript 1; HOXA11-AS: HOXA11 Antisense RNA].

### 4.3. LncRNAs SNPs List in Colorectal Cancer (CRC), Their Role and Mechanism of Action (Table 6)

As listed in Table 6, researchers found lncRNA MALAT1 SNPs rs619586, rs664589, and rs1194338 are associated with CRC risk through binding of various TFs [148]. rs67085638 in lncRNA CCAT1 increases risk of CRC, while rs7013433 is related to CRC late clinical stage [149]. On the other hand, rs2470151 (CT/TT genotype) in lncRNA RP11-108K3.2 could decrease the risk of CRC [150]. Linc-ROR SNP rs1942347 was associated with CRC large tumor size and mortality [151]. rs2839698 in lncRNA H19 associated with increased CRC risk, changing promotor activity and H19 function [152]. Moreover, H19 SNPs rs4930101, rs11042170, and rs27359703 were associated with increased risk of CRC [153]. HOTTIP is an antisense lncRNA that is upregulated in different tumors including CRC. SNPs rs3807598, rs2067087, and rs17427960 in HOTTIP increase susceptibility to CRC, through affecting Transcription Factor Binding Sites (TFBSs) according to SNP function prediction [154]; however, rs1859168 regulates lncRNA gene expression [155]. A positive association between rs55829688 SNP in GAS5 and CRC risk reduced GAS5 expression by altering the TF YY1′s affinity for GAS5 [156]. It has been reported that SNP rs2632159 in lncRNA PCAT1 can elevate the risk of CRC [157]. It has been demonstrated that lncRNA PUNISHER “AGAP2-AS1” has a role in inducing CRC cell proliferation, epithelial-to-mesenchymal transition, and enhancement of CRC cells’ chemoresistance to gemcitabine. PUNISHER is associated with elevated risk of CRC, tumor relapse, and short survival time, via rs12318065. This SNP could modify regulatory motifs such as MRG1, Sin3Ak-20 disc6, and HOXA9 1 that have been found to be linked to CRC, or affecting binding with the TFs POL2, ZNF263, and STAT1, associated with carcinogenesis [158]. It has been demonstrated that lncRNA MAGI2-AS3 acts as a tumor suppressor in many cancers; however, it helps in CRC progression. An increased risk for CRC is due to SNP rs7783388 in lncRNA MAGI2-AS3, via influencing the binding ability of glucocorticoid receptor (GR) to the MAGI2-AS3 promoter [159]. SNHG16 is a lncRNA located on human chromosome 17q25.1, upregulated in BC, bladder cancer, and CRC, where it affects the expression of genes associated with lipid metabolism. SNP rs7353 in lncRNA SNHG16 suppresses CRC, while rs8038 and rs15278 increase this risk [160]. Via binding to miR-4658 and impairing the expression of lncRNA CCSlnc362 “RP11-362K14.5” in CRC cells [161], SNP rs1317082 can protect against CRC. LincRNA Papillary Thyroid Carcinoma Susceptibility Candidate 3 (PTCSC3) is considered as a tumor-suppressor in thyroid cancer and glioma [162]. SNP rs944289 in PTCSC3 decreases CRC risk [163]. rs1456315 in lncRNA prostate cancer non-coding RNA (PRNCR1) increases CRC risk [164]. LncRNA MEG3 SNP rs7158663 elevates CRC risk [165]. By losing binding of miR-128-3p to LAMC2-1:1, rs2147578 in lncRNALAMC2-1:1 increased CRC risk [166]. SNP rs7958904 in lncRNA HOTAIR is associated with both CRC mortality and incidence [167]. LncRNA UCA1 is upregulated in different types of cancer including CRC, BC, bladder cancer, NSCLC, esophageal cancer, and TSCC. SNP rs12982687 in lncRNA UCA1 was proved to affect UCA1’s binding to miR-873-5p and HIF-1 signaling, resulting in a contribution to the progression of smoking-triggered CRC [168]. SNPs rs6983267 at 8q24 and HULC rs7763881 may serve as genetic indicators of a propensity towards CRC and are correlated with CCAT2 and HULC expression, respectively [169].

TINCR lncRNA SNPs rs2288947, rs8105637 are associated with CRC progression and susceptibility. The G allele of SNP rs2288947 was associated with decreased CRC risk, while the A allele of SNP rs8105637 was associated with increased CRC risk. The mechanism involves five motifs, Nanog disc3, CTCF disc9, Rad21 disc10, SP1 disc3, and SMC3 disc3, which may be affected by rs2288947. rs8105637 affects TCF12 and PITX2 expression linked to carcinogenesis [170].

**Table 6:** LncRNA SNPs list in Colorectal Cancer (CRC), their mechanism of action and role in CRC.

LncRNA List	SNPs List	Mechanism of Action [Ref.]	Role in CRC	Type of Samples Used in the Study
MALAT1	rs619586, rs664589, rs1194338	Affecting binding of TFs [146]	Associated with risk	Extracted DNA from human blood samples
CCAT1	rs67085638	- [149]	Increases risk	
	rs7013433	- [147]	Related to CRC late clinical stage	
RP11-108K3.2	rs2470151	- [150]	Decreased risk with CT/TT genotype	
LINC-ROR	rs1942347	- [151]	Associated with large tumor size and mortality	Extracted DNA from FFPE tissue samples
H19	rs2839698, rs4930101, rs11042170, rs27359703	rs2839698 change activity of promotor and H19 function [152,153]	Increased risk	Extracted DNA from human blood samples
HOTTIP	rs1859168	Regulates lncRNA gene expression [154,155]	Increased susceptibility	
	rs3807598, rs2067087, rs17427960	Affect TFBSs [154,155]		
GAS5	rs55829688	Reduced GAS5 expression by altering TF YY1′s affinity to GAS5 [154]	Increased risk	
PCAT1	rs2632159	- [155]	Increased risk	
PUNISHER “AGAP2-AS1”	rs12318065	Modify regulatory motifs; MRG1, Sin3Ak-20_disc6, HOXA9_1, affect TFs binding POL2, ZNF263, and STAT1 [156]	Elevated risk, tumor relapse and short survival time	Extracted DNA from FFPE tissue samples
MAGI2-AS3	rs7783388	Influencing binding ability of GR to lncRNA promoter [157]	Increased risk	Extracted DNA from human blood samples
SNHG16	rs7353	Influencing lncRNA expression [158]	Suppresses susceptibility	
	rs8038, rs15278		Increased risk	
CCSlnc362 “RP11-362K14.5”	rs1317082	Binding miR-4658 and impairing lncRNA expression [159]	Protection	Human CRC tissues vs. adjacent normal tissues and human CRC cell lines HCT116, DLD-1, SW480, LOVO, HT29, RKO vs. the immortalized human colorectal epithelial cell line FHC
PTCSC3	rs944289	- [163]	Decreased risk	Extracted DNA from human blood samples
PRNCR1	rs1456315	- [164]	Increased risk	
MEG3	rs7158663	- [165]	Increased risk	
LAMC2-1:1	rs2147578	Losing miR-128-3p binding [164]	Increased risk	
HOTAIR	rs7958904	- [165]	Associated with mortality and incidence	
UCA1	rs12982687	Affecting UCA1’s binding to miR-873-5p and HIF-1 signaling [166]	Progression of smoking-triggered CRC	
HULC	rs7763881	Correlated with expression of HULC [167]	Genetic indicator for CRC	
TINCR	rs2288947	Affected motifs; Nanog_disc3, CTCF_disc9, Rad21_disc10, SP1_disc3, and SMC3_disc3 [168]	Allele G associated with decreased risk	
	rs8105637	TCF12 and PITX2 expression linked to carcinogenesis [168]	Allele A associated with increased risk	

[MALAT1: Metastasis Associated Lung Adenocarcinoma Transcript 1; CRC: Colorectal Cancer; CCAT1: Colon Cancer Associated Transcript 1; Linc-ROR: Long Intergenic Non-Protein Coding RNA, Regulator Of Reprogramming; FFFPE: formalin-fixed, paraffin-embedded; HOTTIP: HOXA transcript at the distal tip; TFBSs: Transcription Factor Binding Sites; GAS5: Growth Arrest Specific 5; YY1: Yin Yang 1; PCAT1: Prostate cancer-associated transcript 1; AGAP2-AS1: AGAP2 Antisense RNA 1; MRG1: melanocyte-specific gene-related gene 1; HOXA9: Homeobox protein Hox-A9; POL2: RNA Polymerase 2; ZNF263: Zinc Finger Protein 263; STAT1: Signal transducer and activator of transcription 1; FFPE: Formalin-fixed, paraffin-embedded; MAGI2-AS3: MAGI2 Antisense RNA 3; GR: Glucocorticoid Receptor; SNHG16: Small Nucleolar RNA Host Gene 16; PRNCR1: Prostate Cancer Associated Non-Coding RNA 1; MEG3: Maternally Expressed 3; HOTAIR: HOX transcript antisense RNA; UCA1: Urothelial cancer associated 1; HIF-1: Hypoxia-inducible factor; CCAT2: Colon Cancer Associated Transcript 2; HULC: Highly upregulated in liver cancer; TINCR: Terminal differentiation-induced non-coding RNA; CTCF: CCCTC-binding factor; SP1: Specificity protein 1; SMC3: Structural Maintenance Of Chromosomes 3; TCF12: Transcription Factor 12; PITX2: Paired Like Homeodomain 2].

### 4.4. LncRNAs SNPs List in Pancreatic Cancer, Their Role and Mechanism of Action (Table 7)

ANRIL’s SNP rs1537373 may enhance pancreatic cancer susceptibility through TFs binding and Cyclin-dependent kinase inhibitor 2B (CDKN2B) expression regulation [171]. Moreover, another study reported SNP rs1412832 in ANRIL may increase PDAC risk. Several target genes are regulated by ANRIL, including CDKN2A and p16, which typically exhibits harmful somatic and germline mutations and dysregulation [172]. SNPs rs4759314 and rs200349340 in HOTAIR lncRNA can increase pancreatic cancer susceptibility. The later SNP affects HOTAIR expression by interfering with binding with miR-29a [173]. Furthermore, the rs7046076 variant in lncRNA structural maintenance of chromosomes 2 (lnc-SMC2-1) increases PDAC risk, interfering with the lncRNA’s ability to bind to miR-1256 [174]. Although LINC00673 is an oncogene in many cancer types including NSCLC, it is a tumor suppressor in Pancreatic cancer [175]. Pancreatic cancer risk increased via creating a binding site for miR-1231 upon the occurrence of SNP rs11655237 in LINC00673, via limiting degradation of protein tyrosine phosphatase non-receptor type 11 (PTPN11) [176]. On the other hand, Hu et al. have found that SNP rs1859168 in HOTTIP may reduce pancreatic cancer susceptibility by suppressing HOTTIP expression [177].

**Table 7:** LncRNAs SNPs list in pancreatic cancer, their mechanism of action and role in pancreatic cancer.

LncRNA List	SNPs List	Mechanism of Action [Ref.]	Role in Pancreatic Cancer	Type of Samples Used in the Study
ANRIL	rs1537373	Affecting TF binding and regulating CDKN2B expression [171]	Increased susceptibility	Extracted DNA from human blood samples
	rs1412832	CDKN2A, p16 exhibit harmful somatic, germline mutations and dysregulation [172]	Increased risk	
HOTAIR	rs4759314	- [173]	Increased susceptibility	
	rs200349340	Interfering with binding of miR-29a [173]		
lnc-SMC2-1	rs7046076	Interfering with binding to miR-1256 [174]	Increased risk	
LINC00673	rs11655237	Binding site for miR-1231 and limits PTPN11 degradation [176]	Increased risk	Extracted DNA from human PDAC tissues vs. adjacent normal tissues
HOTTIP	rs1859168	- [177]	Decreased susceptibility	Extracted DNA from human blood samples

[ANRIL: Antisense non-coding RNA in the INK4 locus, CDKN2B: Cyclin-dependent kinase inhibitor 2B, CDKN2A: Cyclin-dependent kinase inhibitor 2A, HOTAIR: HOX transcript antisense RNA, lnc-SMC2-1: LncRNA Structural Maintenance of Chromosomes 2, PTPN11: Protein Tyrosine Phosphatase Non-receptor type 11, HOTTIP: HOXA transcript at the distal tip].

### 4.5. LncRNAs SNPs List in Hepatocellular Carcinoma, Their Role and Mechanism of Action (Table 8)

SNP rs7958904 in HOTAIR, SNPs rs3931282, rs1134492, and rs10589312 in Plasmacytoma variant translocation 1 (PVT1) and SNP rs84557 in Epidermal growth factor receptor-Antisense RNA 1 (EGFR-AS1) have been linked to the occurrence and prognosis of HCC through affecting their binding to different effector miRs, as listed in Table 7 [178]. HOTTIP SNPs rs2067087, rs17501292, and rs17427960 and rs4102217 in the MALAT1 lncRNA increased HCC susceptibility by the regulation of certain motifs that elevate the expression of these carcinogenic lncRNA [179]. SNP rs2839698 in lncRNA-H19 can predict the risk and prognosis of HCC [180]. LN metastasis and HCC’s increased susceptibility were related to SNP rs9914618 in LINC00673 [181].

**Table 8:** LncRNAs SNPs list in HCC, their mechanism of action and role in HCC.

LncRNA List	SNPs List	Mechanism of Action [Ref.]	Role in HCC
HOTAIR	rs7958904	Binding miR-615-3p [176]	Linked to incidence and prognosis
PVT1	rs3931282, rs1134492, rs10589312	Bind miR-205-5p, 34b-5p, 183-3p, 31-5p [176]	
EGFR-AS1	rs84557	Binding miR-33b-5p [176]	
HOTTIP	rs2067087, rs17501292, rs17427960	Regulation of certain motifs [177]	Increased susceptibility
MALAT1	rs4102217		
H19	rs2839698	- [178]	Prediction of risk and prognosis
LINC00673	rs9914618	- [179]	Increased susceptibility and LN metastasis

DNA was extracted from human blood samples. [HCC: Hepatocellular Carcinoma, HOTAIR: HOX transcript antisense RNA, PVT1: Plasmacytoma variant translocation 1, EGFR-AS1: Epidermal growth factor receptor-Antisense RNA 1, HOTTIP: HOXA transcript at the distal tip, MALAT1: Metastasis Associated Lung Adenocarcinoma Transcript 1, LN: Lymph Node].

Summary of Point 4 “LncRNAs SNPs in different cancer types and their mechanism of action”: SNPs in lncRNAs can increase or decrease the risk of various cancer types such as BC, CRC, HCC, etc., through different mechanisms (summarized in Table 4, Table 5, Table 6, Table 7 and Table 8). For example, SNP rs920778 in HOTAIR increases the risk of BC, while SNP rs3200401 in MALAT1 decreases the risk of BC. These studies could help in the progress of cancer treatments.

## 5. Summary and Conclusions

**Figure 3:**
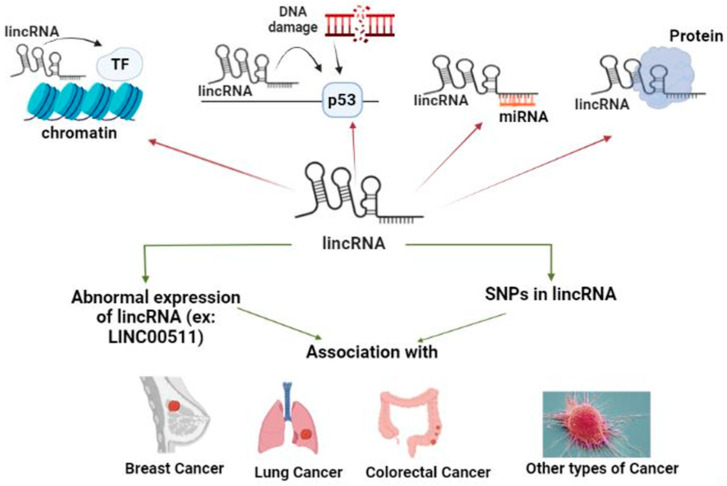
LincRNAs different functions, abnormal expression, and SNPs association with various types of cancer (presented as the review conclusion summary and the graphical abstract.). LincRNAs have different functions, such as having a role in chromatin remodeling and DNA damage repair, in addition to acting as ceRNA and protein scaffold. Abnormal expression of lincRNAs, for example LINC00511, and SNPs in lincRNAs are associated with various types of cancer such as BC, lung cancer, CRC, and other types of cancer.

One abundant class of lncRNAs is lincRNAs, which are involved in various important biological processes. Numerous lincRNAs have been proved to be related to cancer, either being oncogenic, increasing cancer risk, susceptibility, progression, and/or metastasis, or decreasing cancer risk, being tumor suppressors, through different mechanisms of TFs or E2F, signaling pathways, or sponging various miRs. Specifically, LINC00511 has a crucial role in various types of cancer. Different lncRNAs SNPs or particularly LINC00511 SNPs were associated with cancer risk/protection, through distinct pathways, that could be a potential target/hit for cancer treatment as presented in Figure 3. 

Implementing ncRNA measurements in blood liquid biopsy or tumor tissues will be a step toward ncRNA precision health and fulfilling both big pharma’s intention as well as the Sustainable Development Goals’ goal 3 (SDGs #3) (Better Health).

Strengths of the current review study: Our review article covered almost all lincRNAs and their role in various cancer types. LINC RNA constitute promising hit target(s) for the design of chemotherapy treatment for different types of cancer, a step toward ncRNA precision treatment.

Limitation: Pathways of some lincRNAs are still unknown or missing in different in silico/bioinformatic databases and this mandates further studies, in either clinical or in vitro experimental manners, to prove findings.

Future Prospective: More future studies are required to link lincRNAs SNPs variants and haplotypes in different types of cancer, to help pick cancer cases in an early-stage or low-grade, identifying the pre-treatment predictors of response to therapy, ensuring personalized earlier identification, improvement of patients’ survival, complementing the epigenome project, implementing ncRNA measurements in liquid biopsy or tumor tissues, a step toward ncRNA precision. Our research group “Epigenetics studies in Cancer” came into the way, at the advanced biochemistry research lab (ABRL) at the Biochemistry Dept., Faculty of Pharmacy, Ain Shams University, second to extensively studied tumor-suppressor(s) and/or oncogenic gene(s), their SNPs, variants, and haplotypes [182,183,184,185,186,187] in different cancer types.

Sustainability: as an initiative for decoding carcinogenesis from a ncRNA perspective [188], our research group are currently measuring three LINC00511 SNPs variants haplotypes in BC, HCC, and CRC clinical cohorts. Second, their link to multidrug resistance, and/or ce-miRs [189].

## Figures and Tables

**Table 1:** Examples of lncRNAs transcripts ID, their gene ID, strand (+/−) and chromosomal location according to LNCipedia v5.2.

LncRNA Transcripts ID	Gene ID	Chromosome Location (hg38)	Strand
lnc-DAAM2-9:1 to 10	lnc-DAAM2-9	chr6	+
VLDLR-AS1:8 to 10	VLDLR-AS1	chr9	−
LINC01228:1	LINC01228	chr16	−
LINC00951:1 to 6	LINC00951	chr6	−
LINC-ROR:1 to 5	LINC-ROR	chr18	−
LINC-PINT:11 to 81	LINC-PINT	chr7	−
GACAT2:2	GACAT2	chr18	−

[hg38: ID used for Genome Reference Consortium Human Reference 38; chr6: chromosome 6; VLDLR-AS1: VLDLR Antisense RNA 1; LINC01228: Long Intergenic Non-Protein Coding RNA 1228; LINC-ROR: Long Intergenic Non-Protein Coding RNA, Regulator Of Reprogramming; LINC-PINT: Long Intergenic Non-Protein Coding RNA-P53 Induced Transcript; GACAT2: Gastric Cancer Associated Transcript 2; + positive: forward strand; − negative: backward/reverse strand]. accessed on 22 November 2022 and revised on 31 March 2023. https://lncipedia.org/db/search?search_id=lincRNA&search_source=all&search_chromosome=-&search_start=&search_end=&search_class=intergenic&search_keyword=&search_seq=&high_confidence_set=1&search_idhistory_version=4_1&search_idhistory_type=gene&search_idhistory_id=&page=0.

**Table 3:** LINC00511 role in different types of cancer, expression level if upregulated or downregulated, sponging-miR, mechanism of action, role in cancer as oncogene or tumor suppressor.

Cancer Type	Expression	Sponged miR-	Mechanism of Action [Ref.]	Role in Cancer	Type of Samples Used in the Study
CRC	Upregulated	153-5p	HIF-1 activates LINC00511, targeting HIF-1’s 3-UTR and +ve feedback loop [78]	Oncogene	Human CRC tissues vs. paired adjacent non-tumor and CRC cell lines HT29, LOVO, SW620, SW480 vs. normal colon epithelial FHC cell line and athymic BALB/c nude mice for implantation
		-	HNF4 promotes LINC00511 transcription, interaction with EZH2/IL-24 expression downregulation [81]		Human CRC tissues vs. adjacent normal tissues and CRC cell lines HCT116, HT-29, LoVo, SW480, SW620, immortalized human colonic mucosal epithelial cell line NCM460, and female athymic BALB/c mice for implantation
		29c-3p	Upregulation of NFIA [82]		Human CRC tissues vs. normal tissues and CRC cell lines HT-29, HCT8, HCE8693, SW620 vs. normal cell line NCM460
		625-5p	Enhancing WEE1 protein [83]		Human CC tissues vs. normal tissues and human CC cells SW480, SW620, HCT16, HT29 vs. normal cell line NCM460 and male nude mice for implantation
Lung	Upregulated	-	Binding to LSD1 and EZH2 inhibiting LATS2 and KLF2 genes [84]	Oncogene	Human NSCLC tissues vs. adjacent normal tissues and human lung cancer cell lines A549, PC9 and H460 vs. normal human bronchial epithelial cell line BEAS-2B
		-	Binding EZH2 and silencing p57 expression [85]		Human NSCLC tissues vs. adjacent normal tissues and human NSCLC cell lines A549, SK-MES-1, H1299, 95D, H460, H520, H1975, H157, SK-LU-1, SPC-A-1 vs. normal human bronchial epithelial cell line 16HBE
		625-5p	Regulation of PKM2 expression [86]		Human lung adenocarcinoma tissues vs. adjacent normal lung tissues and human lung adenocarcinoma cell lines H1299and A549 vs. human pulmonary epithelial cells BEAS-2B
		195-5p	Upregulation of GCNT3 [87]		Human lung adenocarcinoma tissues vs. adjacent normal tissues and human lung adenocarcinoma cells A549, Calu-3, DV-90, PC-9 vs. human bronchial epithelial cells BEAS-2B
		625-5p	Targeting GSPT leading to progression and invasion of lung cancer cells [88]		Human NSCLC tissues vs. normal tissues and NSCLC cell lines A549, NCIH1299, NCIH1650, NCIH1975, NCIH460 vs. human bronchial epithelial cell line 16HBE
		150-5p	Activating TADA1 leading to progression and migration of lung cancer [89]		Human lung squamous cell carcinoma cell lines SK-MES-1, H226 vs. the human bronchial epidermal cells 16-HBE and TCGA database for LINC00511 expression in lung squamous cell carcinoma tissues
CC	Upregulated	-	Upregulating PLD1 expression, through the transcription factor RXRA leading to progression of the disease [90]	Oncogene	Human CC tissues vs. paracancerous tissues and human CC cells SiHa, CaSki, C33A, ME180, HeLa vs. normal cervical epithelial cells NCECs and mixed gender BALA/C nude mice for implantation
		-	LINC00511 inhibition results in lowering cell viability, inducing apoptosis, regulating Bcl-2, Bax, metalloproteinases 2 and 9, MRP1, P-glycoprotein and cleaved caspase-3, increasing Paclitaxel sensitivity and decreasing proliferation [91]		Human CC tissues vs. adjacent normal tissues and human CC cell line HeLa
		324-5p	Regulation of DRAM1 axis leading to CC progression and invasion of CC cells [92]		Human CC tissues vs. paracancerous tissues and human CC cell lines SiHa, CaSki; HPV-positive vs. C33A; HPV-negative, normal cervical epithelial cells HUCEC, HEK 293T
		497-5p	Regulation of MAPK1 axis leading to progression of the disease [93]		Human CC tissues vs. para-cancerous tissues and human CC cell lines SiHa, HeLa, C4-1, HT-3 vs. normal cervical epithelial cell line End1/E6E7
GC	Upregulated	195-5p	Elevation of SOX4 and EZH2 and repression of PTEN to activate P1_3_/AKT resulting in GC tumorigenesis and stemness [94]	Oncogene	Human GC cell lines AGS, HGC-27, ACP01, SNU-1 vs. normal oesophageal epithelial cell line Het-1A and Male BALB/C nude mice for implantation
		515-p	MAPK signaling pathway leading to GC cells’ invasion and progression of the disease [95]		Human GC tissues vs. normal gastric tissues and human GC cell lines AGS, SGC7901, BGC823, MKN45, MGC803 vs. gastric epithelial cell line GES-1 and BALB/c nude mice for implantation
		124-3p	Regulation of PDK4 resulting in progression of the disease [96]		Human GC cell lines MKN-45, BGC-823, HGC-27, MGC-803 vs. normal gastric epithelial cells GES-1
		625-5p	Targeting NFIX resulting in progression of the disease [97]		Human GC tissues vs. adjacent normal tissues and human GC cells HGC27, BGC823, MGC803, SGC7901 vs. gastric mucosa epithelial cell GES1 and nude mice for implantation
PDAC	Upregulated	29b-3p	Upregulation of VEGFA leading to PDAC stemness [98]	Oncogene	Human pancreatic cancer tissues vs. adjacent normal pancreatic tissues and human pancreatic cancer cell lines PANC-1, MIA PaCa-2, Capan-2, SW1990, ASPC-1, BxPC-3, immortalized human pancreatic ductal epithelial cell line HPDE6 and nude mice for implantation
HCC	Upregulated	424	Promoting progression and metastasis of HCC [99]	Oncogene	Human HCC tissues vs. normal tissues and human HCC cell lines Hep3B, HepG2, SMMC-7721, MHCC97H, Huh7, HCCLM3 vs. normal liver cells LO2
		195	Correlating with EYA1 and promoting HCC progression [100]		Human HCC cell lines SMCC7721, HepG2, Huh7, Hep3B vs. normal hepatocytes L-02 and TCGA database for LINC00511/miR-195/EYA1 expression levels in HCC tissues
		29c	[101]		Human HCC tissues vs. paracancerous tissues and human HCC cell lines MHCC-97H, Huh7, HCC-LM3, Hep3B, MHCC-97L, Huh6 vs. normal hepatocytes LO2
		-	Affecting exosome secretion and invadopodia formation leading to progression of HCC [102]		Human HCC tissues vs. adjacent normal tissues and human cell lines Huh7, Hep3B and female BALB/c nude mice for implantation
GBM	Upregulated	524-5p	Indirectly controlling YB1 and boosting ZEB1, forming + feedback loop leading to migration and invasion of GBM cells [103]	Oncogene	Human GBM tissues vs. normal brain tissues and human GBM cell lines U87, LN229, U251, A172 vs. normal human astrocyte line NHA, HEK 293T and male BALB/c nude mice for implantation
		15a-5p	LINC00511 knockdown and targeting miR-15a-5p/AEBP1 axis result in prevention of GBM [104]		Human GBM tissues vs. adjacent normal tissues and human GBM cell lines T98 G, A172, LN229, U-87MG, U-251MG vs. normal human astrocytes HEB, NHA
		126-5p	Activation of Wnt/β-catenin signaling and Temozolomide resistance [105]		Human GBM tissues vs. adjacent normal tissues and human GBM cell lines U87, A172, U138, U251, U373, LN-18, T98G, HEK293T and female BALB/C nude mice for implantation
OS	Upregulated	618	Boosting MAEL expression and promoting progression of the disease [106]	Oncogene	Human OS tissues vs. adjacent normal tissues and human OS cell lines MG-63, HOS, Saos-2 and 143B vs. osteoblast cell line hFOB 1.19 and male BALB/c nude mice for implantation
		185-3p	Regulation of E2F1 expression leading to progression and invasiveness of OS [107]		Human OS tissues vs. adjacent normal tissues and human OS cell lines SW1353, U2OS
		765	Promoting APE1 and progression of OS [108]		Human OS tissues vs. adjacent normal tissues and human OS cell lines MG-63, Saos-2, U2OS, HOS vs. normal osteoblast cell line NHOst
	Downregulated	-	Inhibition of proliferation and increasing apoptosis of OS cells and tumor cell necrosis rate (TCNR) [109]	Tumor suppressor	Human OS tissues vs. adjacent normal tissues and human OS cell lines MG-63, U-2OS, Saos-2, and HOS vs. hFOB1.19, 293T cells and male BALB/c-nude mice for implantation
BC	Upregulated	150	Regulation of MMP-13 and inducing BC cell migration [110]	Oncogene	Human BC tissues vs. normal breast tissues and MDA-MB-231 cell line vs. MCF-7 cells
		-	DNA hypomethylation induce LINC00511; Wnt10A, E2F2, TGFA, MET upregulation [111]		Human BC tissues vs. normal tissues and MDA-MB-231, MCF-7, T47D, MDA-MB-468, MCF-10a
		185-3p	targeting E2F1 protein to bind Nanog promotor region activation [80]		Human BC tissues vs. adjacent normal tissues and human BC cell lines MDA-MB-468, MDA-MB-231, MDA-MB-453 and MCF-7 vs. MCF-10A and male null mice for implantation
		185-3p	Increasing BC cells expression, transcription control of downstream genes [42]		Human blood samples
		-	[112]		Human BC tissues vs. adjacent normal tissues and BC cell lines MDA-MB-453, MCF-7, UACC812, T47D
		-	Accelerating G1/S transition, apoptosis suppression and enhancement of -ve ER BC cell growth [113]		MCF7, UACC-812, MDA-MB-231, T47D and pathogen-free female athymic BALB/c mice for implantation
		29c	LINC00511 downregulation enhances Paclitaxel cytotoxicity by regulating miR-29c/CDK6 axis [114]		Human BC tissues vs. adjacent normal tissues and MDA-MB-231, MCF-7, Hs-578T, T47D, immortalized breast epithelial cell line MCF-10A
		185	Regulating STXBP4 expression and promoting BC recurrence and radio-resistance [115]		Human BC tissues vs. adjacent normal tissues and MDA-MB-231, MDA-MB-436, MDA-MB-361, MCF-7, in addition to breast epithelial cell MCF-10A and nude mice for implantation

N.B. The approach of all the studies was to knockdown LINC00511. [CRC: Colorectal Cancer; HIF-1: Hypoxia Inducible Factor 1; HNF4: Hepatocyte Nuclear Factor 1; EZH2: Enhancer of zeste homolog 2; IL-24: Interleukin 24; NFIA: Nuclear Factor 1 A; LSD1: Lysine-Specific Demethylase 1; LATS2: Large Tumor Suppressor Kinase 2; KLF2: KLF Transcription Factor 2; PKM2: Pyruvate kinase M2; GCNT3: glucosaminyl (N-acetyl) transferase 3; TADA1: Transcriptional Adaptor 1; CC: Cervical Cancer; PLD1: Phospholipase D1; RXRA: Retinoic X Receptor Alpha; Bcl-2: B-cell lymphoma 2; Bax: Bcl-2 Associated X-protein; MRP1: Multidrug Resistance Protein 1; DRAM1: DNA Damage Regulated Autophagy Modulator 1; HPV: Human papillomavirus; MAPK1: Mitogen-Activated Protein Kinase 1; GC: Gastric Cancer; SOX4: SRY-box transcription factor 4; PTEN: Phosphatase and tensin homolog; PDK4: Pyruvate dehydrogenase lipoamide kinase isozyme 4; NFIX: Nuclear Factor 1/X gene; PDAC: Pancreatic Ductal Adenocarcinoma; VEGFA: Vascular Endothelial Growth Factor Alpha; HC: Hepatic Cancer; GBM: Glioblastoma; YB1: Y box binding protein 1; ZEB1: Zinc finger E-box-binding homeobox 1; AEBP1: AE Binding Protein 1; OS: Osteosarcoma; MAEL: Maelstrom spermatogenic transposon silencer; E2F1: E2F transcription factor 1; APE1: Apurinic/Apyrimidinic endonuclease 1; BC: Breast Cancer; MMP-13: Matrix Metallopeptidase 13; Wnt10A: Wnt family member 10A; E2F2: E2F transcription factor 2; TGFA: Transforming growth factor alpha; ER: Estrogen Receptor; CDK6: Cyclin-Dependent Kinase 6; STXBP4: Syntaxin Binding Protein 4].

## Data Availability

Not applicable.

## Abbreviations

AEBP1	AE Binding Protein 1
AGAP2-AS1	AGAP2 Antisense RNA 1
ANRIL	Antisense non-coding RNA in the INK4 locus
APE1	Apurinic/apyrimidinic endonuclease 1
AQP3	Aquaporin 3
AQP4-AS1	AQP4 Antisense RNA 1
AS	Antisense
Bax	Bcl-2 Associated X-protein
BC	Breast Cancer
Bcl 2	B-cell lymphoma 2
BRCA1	Breast Cancer antigen 1
BRCA2	Breast Cancer antigen 2
CASC15	Cancer Susceptibility 15
CC	Cervical cancer
CCAT1	Colon Cancer Associated Transcript 1
CCAT2	Colon Cancer Associated Transcript 2
CCND2	Cyclin D2
CDK6	Cyclin-dependent kinase 6
CDKN2A	Cyclin-dependent kinase inhibitor 2A
CDKN2B	Cyclin-dependent kinase inhibitor 2B
CDKN2B-AS1	Cyclin-dependent kinase inhibitor 2B antisense RNA 1
ceRNA	competitive endogenous RNA
chr6	chromosome 6
CRC	Colorectal Cancer
CREB	cAMP response element-binding protein
CSCs	Cancer Stem Cells
CTCF	CCCTC-binding factor
DDR	DNA Damage Repeat
DRAM1	DNA Damage Regulated Autophagy Modulator 1
DSB	Double Strand Break
E2F1	E2F Transcription Factor 1
E2F2	E2F Transcription Factor 2
EGFR	Epidermal growth factor receptor
EGFR-AS1	Epidermal growth factor receptor-Antisense RNA 1
EMT	Epithelial-Mesenchymal Transition
ENO1	Enolase 1
ER	Estrogen Receptor
EREG	Epiregulin
EYA1	Eyes absent homolog 1
EZH2	Enhancer of zeste homolog 2
FFPE	Formalin-fixed, paraffin-embedded
FOXM1	Forkhead box M1
G3BP1	G3BP Stress Granule Assembly Factor 1
GACAT2	Gastric Cancer Associated Transcript 2
GAS5	Growth Arrest Specific 5
GBM	Glioblastoma
GC	Gastric Cancer
GCNT3	Glucosaminyl (N-acetyl) transferase 3
GOLPH3	Golgi Phosphoprotein 3
GR	Glucocorticoid Receptor
GWAS	Genome Wide Association Studies
HC	Hepatic Cancer
HCC	Hepatocellular Carcinoma
HCV	Hepatitis C Virus
HER-2	Human epidermal growth factor receptor 2
hg38	ID used for Genome Reference Consortium Human Reference 38
HIF-1	Hypoxia Inducible Factor 1
HNF4	Hepatocyte Nuclear Factor 4
hnRNP-K	heterogeneous nuclear ribonucleoprotein-K
HOTAIR	HOX transcript antisense RNA
HOTTIP	HOXA transcript at the distal tip
HOXA9	Homeobox protein Hox-A9
HOXA11-AS	HOXA11 Antisense RNA
HPV	Human papillomavirus
HULC	Highly upregulated in liver cancer
IL-24	Interleukin 24
KDM2A	Lysine-specific demethylase 2A
KLF2	KLF transcription factor 2
LATS2	Large Tumor Suppressor Kinase 2
LINC00511	Long intergenic non coding RNA 00511
LINC-PINT	Long Intergenic Non-Protein Coding RNA, P53 Induced Transcript
lincRNAs	Long intergenic non-coding RNAs
lincRNA-BC2	Long intergenic non-coding RNA-Breast Cancer 2
Linc-ROR	Long Intergenic Non-Protein Coding RNA, Regulator Of Reprogramming
LN	Lymph Node
LncRNA	Long non coding RNA
lnc-SMC2-1	Long non coding RNA structural maintenance of chromosomes 2
LSCC	Laryngeal squamous cell carcinoma
LSD1	Lysine-specific demethylase 1
MAEL	Maelstrom Spermatogenic Transposon Silencer
MAGI2-AS3	MAGI2 Antisense RNA 3
MALAT1	Metastasis Associated Lung Adenocarcinoma Transcript 1
MAPK1	Mitogen-activated protein kinase 1
MEG3	Maternally Expressed 3
MIR2052HG	MIR2052 Host Gene
miR	Micro-RNA
miRNA	Micro-RNA
MMP13	Matrix Metallopeptidase 13
MRG1	Melanocyte-specific gene-related gene 1
mRNA	Messenger ribonucleic acid
MRP1	Multidrug resistance protein 1
NEAT1	Nuclear Enriched Abundant Transcript 1
NFIA	Nuclear Factor 1 A
NFIX	Nuclear factor 1/X gene
NK	Natural Killer
NKD2	Naked cuticle homolog 2
NSCLC	Non-small cell lung cancer
OS	Osteosarcoma
PC	Pancreatic Cancer
PCAT-1	Prostate Cancer Associated Transcript-1
PRC2	Polycomb Repressive Complex 2
PDAC	Pancreatic ductal adenocarcinoma
PDK4	Pyruvate dehydrogenase lipoamide kinase isozyme 4
PHOX2B	Paired-like homeobox 2b
PITX2	Paired Like Homeodomain 2
PKM2	Pyruvate kinase M2
PLD1	Phospholipase D1
Pol ll	RNA Polymerase ll
PR	Progesterone Receptor
PRNCR1	Prostate Cancer Associated Non-Coding RNA 1
PS	Protein Scafold
PTPN11	Protein Tyrosine Phosphatase Non-receptor type 11
PTCSC3	Papillary Thyroid Carcinoma Susceptibility Candidate 3
PTEN	Phosphatase and tensin homolog
PVT1	Plasmacytoma variant translocation 1
RPIseq	RNA-Protein Interaction Prediction
RNA	Ribo Nucleic Acid
RUNX3	RUNX Family Transcription Factor 3
RXRA	retinoic X receptor alpha
SDGs #3	Sustainable Development Goals; goal 3
SMC3	Structural Maintenance Of Chromosomes 3
smORFs	Small Open Reading Frames
SNHG16	Small Nucleolar RNA Host Gene 16
SNPs	Single Nucleotide Polymorphisms
SOX2OT	SOX2 overlapping transcript
SOX4	SRY-box transcription factor 4
SP1	Specificity protein 1
SR	Serine/Arginine
SRA	Steroid Receptor RNA Activator
ST5	Suppression of tumorigenicity 5
STXBP4	Syntaxin Binding Protein 4
STAT1	Signal transducer and activator of transcription 1
TADA1	Transcriptional Adaptor 1
TASs	Trait-associated SNPs
TCF12	Transcription Factor 12
TCNR	Tumor cell necrosis rate
TFs	Transcription factors
TFBSs	Transcription Factor Binding Sites
TGFA	Transforming growth factor alpha
TINCR	Terminal differentiation-induced non-coding RNA
TNBC	Triple Negative Breast Cancer
TPM	Transcripts per million
TSCC	Tongue Squamous Cell Carcinoma
UCA1	Urothelial cancer associated 1
VEGFA	Vascular endothelial growth factor A
VLDLR-AS1	VLDLR Antisense RNA 1
Wnt	Wingless-INT
Wnt10A	Wnt Family Member 10A
Xist	X-inactive specific transcript
YB1	Y box binding protein 1
YY1	Yin Yang 1
ZEB1	Zinc finger E-box-binding homeobox 1
ZNF263	Zinc Finger Protein 263
